# Advances and Prospects in Biomaterials for Intervertebral Disk Regeneration

**DOI:** 10.3389/fbioe.2021.766087

**Published:** 2021-10-22

**Authors:** Chunxu Li, Qiushi Bai, Yuxiao Lai, Jingjing Tian, Jiahao Li, Xiaodan Sun, Yu Zhao

**Affiliations:** ^1^ Department of Orthopaedics, Peking Union Medical College Hospital, Peking Union Medical College and Chinese Academy of Medical Sciences, Beijing, China; ^2^ Centre for Translational Medicine Research and Development, Shenzhen Institute of Advanced Technology, Chinese Academy of Sciences, Shenzhen, China; ^3^ Medical Science Research Center, Peking Union Medical College Hospital, Peking Union Medical College and Chinese Academy of Medical Sciences, Beijing, China; ^4^ State Key Laboratory of New Ceramics and Fine Processing, School of Materials Science and Engineering, Tsinghua University, Beijing, China

**Keywords:** biomaterial, intervertebral disc, degeneration, nucleus pulposus, anulus fibrosus, cartilage endplate

## Abstract

Low-back and neck-shoulder pains caused by intervertebral disk degeneration are highly prevalent among middle-aged and elderly people globally. The main therapy method for intervertebral disk degeneration is surgical intervention, including interbody fusion, disk replacement, and diskectomy. However, the stress changes caused by traditional fusion surgery are prone to degeneration of adjacent segments, while non-fusion surgery has problems, such as ossification of artificial intervertebral disks. To overcome these drawbacks, biomaterials that could endogenously regenerate the intervertebral disk and restore the biomechanical function of the intervertebral disk is imperative. Intervertebral disk is a fibrocartilaginous tissue, primarily comprising nucleus pulposus and annulus fibrosus. Nucleus pulposus (NP) contains high water and proteoglycan, and its main function is absorbing compressive forces and dispersing loads from physical activities to other body parts. Annulus fibrosus (AF) is a multilamellar structure that encloses the NP, comprises water and collagen, and supports compressive and shear stress during complex motion. Therefore, different biomaterials and tissue engineering strategies are required for the functional recovery of NP and AF based on their structures and function. Recently, great progress has been achieved on biomaterials for NP and AF made of functional polymers, such as chitosan, collagen, polylactic acid, and polycaprolactone. However, scaffolds regenerating intervertebral disk remain unexplored. Hence, several tissue engineering strategies based on cell transplantation and growth factors have been extensively researched. In this review, we summarized the functional polymers and tissue engineering strategies of NP and AF to endogenously regenerate degenerative intervertebral disk. The perspective and challenges of tissue engineering strategies using functional polymers, cell transplantation, and growth factor for generating degenerative intervertebral disks were also discussed.

## Introduction

According to research statistics, 80% of people worldwide have experienced low-back pain in their lifetime (DALYs and Collaborators, 2017). This kind of pain, in severe cases, can radiate to the whole lower limbs, seriously affecting people’s quality of life and work ability while causing a huge medical burden; this disease has become a global social and economic problem (March et al., 2014), and the main cause of this problem is intervertebral disk degeneration (Hoy et al., 2015). For this disease, the current mainstream treatment can be divided into nonsurgical and surgical treatments. Among them, non-surgical treatment methods mainly include drug and physical therapies. The prevailing view is that the causes of low-back pain caused by disk degeneration include an acidic environment caused by local inflammation, nerve root compression due to nucleus pulposus (NP) herniation or disk collapse, and ectopic sensory nerve fibers and blood vessels growing into the annulus and NP (Bailey et al., 2013; Binch et al., 2015; Lama et al., 2018). However, the most important reason is the local inflammatory environment, which is why low-back pain can be effectively ameliorated in the early stage of intervertebral disk degeneration using only NSaids, although there is still no NP herniation. Physical therapy is also thought to be effective in improving low-back pain caused by disk degeneration, and some animal studies have elucidated some of the mechanisms involved. Gawri et al. found that moderate exercise can boost the synthesis of intervertebral disk cells, but excessive exercise can break them down and promote inflammation (Gawri et al., 2014). Gullbrand et al. found that circulating compression and stretching of intervertebral disks can improve the viability of intervertebral disk cells by increasing nutrient transport, thus achieving the effect of physical therapy (Gullbrand et al., 2015). Although some studies have proven the effectiveness of drug and physical therapies, it is difficult for these nonsurgical treatments to achieve significant effects in some patients with severe degeneration because of the obvious inflammatory environment and irreversible cell senescence. At present, for patients with severe degeneration, surgical treatment is still the first choice. The mainstream surgical methods are minimally invasive lumbar discectomy and lumbar open surgery. Although surgery can relieve the symptoms of low back pain, there are problems of long recovery time, high treatment cost, and the possibility of complications such as adjacent segment degeneration and pain recurrence (Ghiselli et al., 2004; Eliasberg et al., 2016; Li et al., 2021). In general, the current mainstream treatment is aimed at the symptoms of treatment but cannot effectively restore the function of the intervertebral disk; therefore, how to restore the original biological and physical function of the intervertebral disk is the focus of scientists’ efforts. Currently, several methods to promote disk regeneration have been reported, including total disk replacement (Takeoka et al., 2020), cell therapy (Hu et al., 2018; Peredo et al., 2020), and gene therapy (Feng et al., 2015; Feng et al., 2020). These new treatments, designed to fully restore or mimic original disk function, show great potential for development. Unlike most reviews, this study first reviews intervertebral disk anatomy and the pathophysiological process of intervertebral disk degeneration, and based on this process, introduces the body’s requirements for regenerative materials. Then, we discussed how the hot materials for regeneration of NP, AF, and cartilage endplates discovered by researchers in recent years are working towards this demand. At the same time, we also analyzed the gap between the current research status and the ideal goal, and proposed that the intervertebral disk should be regarded as an integral movement unit to study its materials in the future, and also briefly introduces the research progress of the overall intervertebral disk regeneration strategy. At present, most review publications of the intervertebral disk regeneration choose to focus on one of the nucleus pulposus or the AF. In comparison, this study can better help researchers to comprehensively understand the materials’ hotspots and provide help for exploring the overall regeneration strategy of intervertebral disk.

## Intervertebral Disk Structure and Function

**FIGURE 1:**
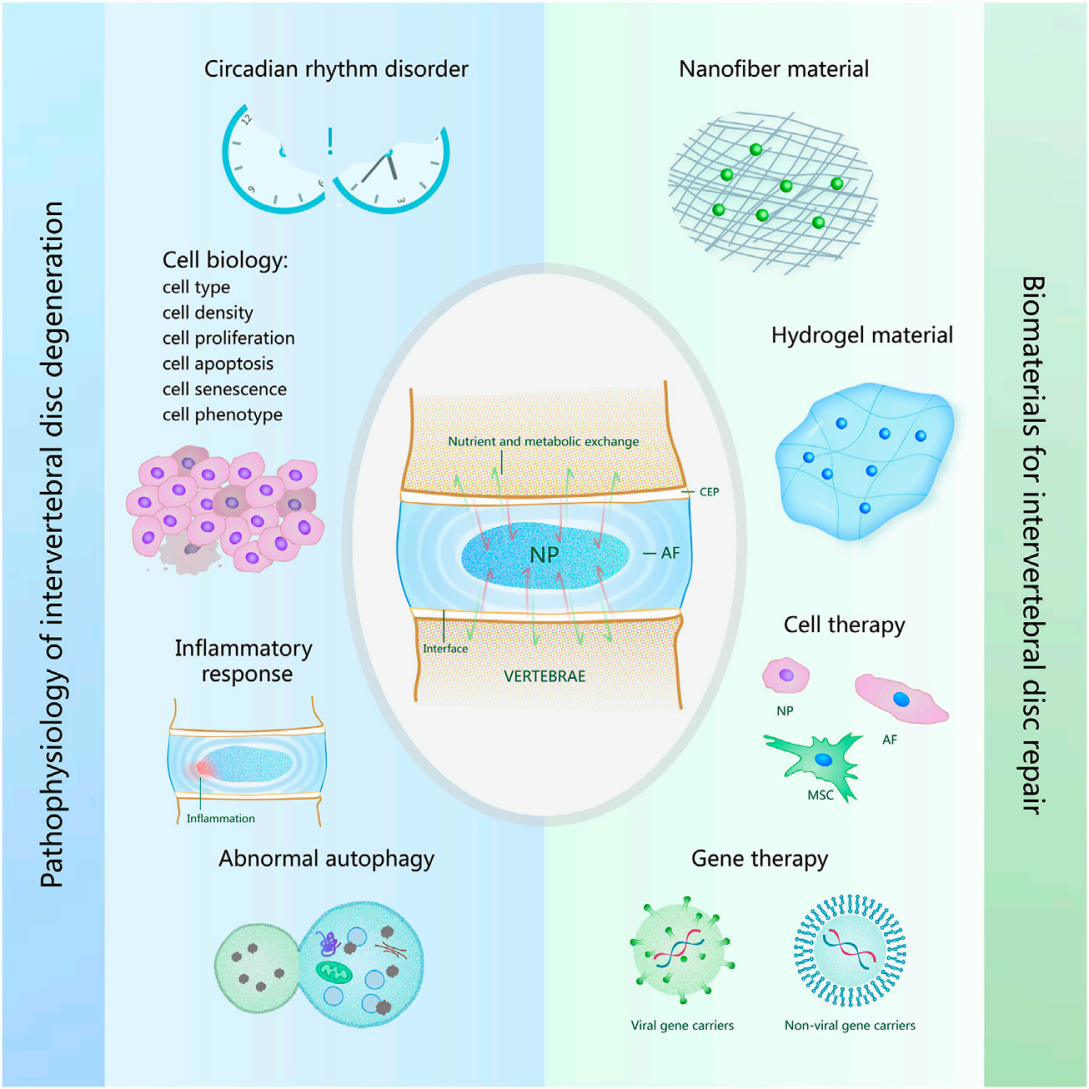
Overview of pathophysiology of intervertebral disk degeneration and biomaterials for intervertebral disk repair. The pathophysiological causes of intervertebral disk degeneration shown on the left of the figure include circadian rhythm disorder, cell biology, inflammatory response, abnormal autophagy. The biomaterials for intervertebral disk repair shown on the right of the figure include nanofiber material, hydrogel material, biomaterials for cell therapy and gene therapy.

The spinal column is one of the most important bone structures in the human body. It protects the spinal cord, supports body weight, slows impact, and allows flexible movement of the trunk. The spine comprises vertebral bones and intervertebral disks (Figure 1). Intervertebral disks, joints between vertebral bones, ligaments, and muscles around them, constitute the most basic motion units of the spine, and intervertebral disks play the most important role in this process. Although many studies have explored intervertebral disk regeneration, its various structure are discussed separately. However, since a unified movement of any part of the overall structure and function of the anomalies is likely to induce the occurrence of low-back pain, we need to understand the various parts of physiology, anatomy, and pathophysiology characteristics, look for differences and commonness among components. It will be convenient to formulate a perfect regeneration plan for the whole structure.

The disk comprises a gelatinous NP, a fibrosus ring surrounding the NP, and a cartilaginous endplate next to the bones of the upper and lower vertebrae, which together bear the complex load caused by compression, extension, bending, and rotation of the spine (Roberts et al., 2006). Although these structures cooperate to accomplish the same task, their functions, compositions, and properties quite differ, and even the components of the inner and outer annulus fibrosus (AF) differ.

NP is located at the disk center; its composition slightly changes with age, and it mainly comprises water, type collagen Ⅱ and elastic protein fiber, protein, polysaccharide, and notochord cells. As aging progresses, notochord cells completely degrade and are replaced by a sample of cartilage cells; concurrently, the collagen fibers begin to pair together; thus, NP by liquid becomes cartilage. However, water still occupies about 85% of the volume, making it viscoelastic and having compressive deformation resistance. NP plays an anisotropic uniformity in absorbing and diffusing stress, and diffuses the pressure to the AF through deformation, thus alleviating the impact on the lower vertebral body (Iatridis et al., 1997). Chondroid cells are metabolically active cells in NP and can synthesize extracellular matrix (ECM) components, such as collagen and proteoglycan. It plays a role in maintaining the balance of the internal environment and is the main element in preventing intervertebral disk degeneration (Akkiraju and Nohe, 2015). Elastin fibers and proteoglycan synergize to maintain collagen activity and restore disk size and shape (Mouw et al., 2014).

AF is a kind of acellular and vascularless structure, mainly including fibrochondrocytes and chondrocytes, which are like fibroblasts in morphology. Its ECM components transition smoothly from inside to outside. The outer layer comprises mainly type I collagen, while the inner layer primarily comprises type II collagen (Guerin and Elliott, 2007). Type II collagen is hypoxic, hydrophilic, and proteoglycan rich, which is consistent with the physics of the hard outer layer and soft inner layer of the AF, which act as the outer layer to bind the NP during bending and twisting. The inner layer binds the NP during axial compression (Eyre and Muir, 1976). From a morphological viewpoint, the AF comprises 15–25 concentric annular lamella, constituting mainly neatly arranged bundles of collagen fibers. The direction of these collagen fibers is 30° to the horizontal plane (Humzah and Soames, 1988). These layers are filled with an interlaminar matrix comprising elastic fibers, cells, water, lipids, and proteoglycans. Proteoglycans including aggrecan, lubricating oil, GAGs, biglycan, decorin, perlecan, versican, etc., are responsible for lubrication and hydration. The cell morphology in the interlaminar matrix varies, from round to spindle and from center to periphery, which are related to the direction and density of elastic fibers, and the direction and magnitude of the load on the adjacent layers. The interlaminar stroma between the different layers is interconnected, connected by dividing boundaries, and comprises a dense elastic fibrosus structure consistent with the AF role in resisting NP expansion.

The cartilaginous endplate is a thin hyaline layer of cartilage, approximately 0.6–1.2 mm thick, with the thinnest and most porous central region (Zehra et al., 2015; Berg-Johansen et al., 2018). Regarding composition, the area adjacent to the cartilage endplate and AF has higher collagen, lower proteoglycan, and lower water contents than the NP (Roberts et al., 1989). Although the AF and the NP and cartilage endplate are anatomically distinct, their fine structures cross each other. The collagen fibers in the AF close to the NP are continuous with the collagen fibers in the cartilage endplate, which helps to reduce excessive concentrations of stress, such as disk stretching, compression, and shearing, and prevent irreversible disk damage (Berg-Johansen et al., 2018). The cartilaginous endplate also acts as a medium for transferring forces in multiple directions between the disk and vertebral body. The cartilage endplate distributes the hydrostatic pressure generated by the NP evenly on the surface of the vertebral body to prevent the NP from expanding locally into the vertebral body. In addition, since the intervertebral disk is the largest vascularless structure in the human body, the blood vessels in the vertebral body are its main source of nutrition, and the capillary network formed by the aorta through all branches forms a capillary loop at the junction of the cartilage endplate and the intervertebral disk (Ashinsky et al., 2021). Therefore, the cartilage endplate is considered the main way for nutrition and waste to be transported to the vertebral body (Figure 1). With aging and degeneration, the cartilage endplate will undergo some composition changes, which will reduce permeability and limit the transportation of nutrients, and is considered one of the reasons for intervertebral disk degeneration (Schroeder et al., 2017).

## Pathophysiology of Intervertebral Disk Degeneration

The height and internal osmotic pressure of intervertebral disks will change under load bearing and resting states (Menon et al., 2021), which is mainly caused by day and night activities and the rest of human body. Interstitial fluid also flows in response to this differential pressure change, resulting in nutrient and metabolic exchanges to maintain intervertebral disk homeostasis (van der Veen et al., 2007). Disruption of circadian rhythms, however, may increase the risk of disk degeneration (Figure 1) (Dudek et al., 2017). Most intervertebral disk cells are nourished by capillaries from the vertebral body. With the deterioration of the intervertebral disk, bone marrow cavity occlusion leads to loss of contact between capillaries and cartilage endplate, and calcification of cartilage endplate increases (Benneker et al., 2005; Hristova et al., 2011).

Intervertebral disk degeneration can induce various cell biological changes, including cell type changes, cell density changes, cell apoptosis, cell proliferation, cell senescence, and cell phenotype changes (Figure 1) (Zhao et al., 2007). Notochord cells are a kind of cell existing in the early stage of human development. As humans grow, notochord cells are replaced by NP cells, suggesting that the onset of intervertebral disk degeneration may be related to the disappearance of notochord cells (McCann et al., 2012). Induction of pluripotent stem cells into notochord cells by injection has also been shown to reduce intervertebral disk degeneration in pig models (Sheyn et al., 2019). The density of NP cells in degenerated disks decreased compared to normal disk tissue (Liu C. et al., 2020). Since excessive mechanical load can induce apoptosis of NP-derived stem cells, some people try to delay intervertebral disk degeneration by anti-apoptosis (He et al., 2021), and promoting NP cell proliferation has also been proven to inhibit intervertebral disk degeneration (Cui et al., 2020). In recent years, with the deepening of research, the phenotypic characteristics of NP cells and the relationship between cell senescence and intervertebral disk degeneration have gradually been considered (Choi et al., 2015; Zhang Y. et al., 2020).

As aging increases, the occurrence of intervertebral disk degeneration is more and more likely (Cheung et al., 2009), and the most common clinically related symptom is low-back pain. According to existing studies, the cause of pain is most likely to be inflammation (Figure 1) (Lyu et al., 2021). Tessier et al. found significant pro-inflammatory pathway changes and age-related disk degeneration in a mouse that was deficient in a protein associated with embryonic disk development (Tessier et al., 2020). Chen et al. found that melatonin can delay the progression of disk degeneration and relieve associated low-back pain by studying its anti-inflammatory effect *in vivo* (Chen et al., 2020).

Recent studies have suggested that intervertebral disk degeneration is also associated with autophagy inhibition (Figure 1) (Lan et al., 2021). Autophagy plays a role in maintaining the homeostasis of the internal environment (Mizushima, 2007). It can meet metabolic requirements through lysosomal degradation and recovery of cell products, and protect cells by removing damaged organelles and misfolded proteins. Autophagy is also associated with other factors that may affect intervertebral disk degeneration. Circadian rhythms may maintain appropriate autophagy to prevent disk degeneration, while abnormal circadian rhythms may induce excessive autophagy and autophagy dysfunction to cause disk degeneration (Zhang T.-W. et al., 2020). Chen et al. found that by triggering autophagy, cell senescence and apoptosis could also be decreased, ultimately improving intervertebral disk degeneration (Chen et al., 2018). In subsequent studies, many people have reduced cell senescence and apoptosis by upregulating autophagy, thus maintaining intervertebral disk repair and delaying degeneration (He et al., 2021; Hu et al., 2021).

## Biomaterials for Nucleus Pulposus Repair

### Nanofiber Material

The disk function depends on NP flexibility and AF toughness. Nanofiber material is a new type of biological material manufactured by stretching method, template synthesis, self-assembly, microphase separation, electrostatic spinning and other methods on the basis of various artificial polymers. Among them, the electrospinning method is widely used in the preparation of medical materials due to its advantages of simple operation, wide application range, and relatively high production efficiency. Because of its highly adjustable shape and structure, it often has excellent mechanical properties. Therefore, how to improve the biological stability of such materials is the focus of attention of researchers. Polyurethane (PU) series of biomaterials can adjust elasticity by changing the composition of monomer units and the block size of different monomers in the polymer chain. Among them, polycarbonate (PC) PU shows better biological stability and can be used as a good material to replace the NP and repair intervertebral disk. A dual-phase PU stent comprising a core material with rapid swelling properties and a flexible electrospinning shell has been prepared and implanted into bovine intervertebral disks, restoring the mechanical properties of enucleated disks and showing the potential to delay further degeneration of natural disk tissue (Li et al., 2016). As cell behavior is affected by ECM characteristics, biomaterials that mimic ECM characteristics are generally beneficial to cell growth (Nesti et al., 2008; Kaur and Roy, 2021), and nanofiber scaffolds have unique physical characteristics that provide favorable cell-matrix cues to enhance cell activities (Figure 1) (Li et al., 2006). Zhang et al. developed a new type of nanofiber sponge microspheres (NF-SMS) with interconnected pore structure, which mimics the ECM protein fiber (Zhang et al., 2015), and has been proven to help mesenchymal stem cell adhesion, proliferation, and np-like differentiation, and the interconnected pores can effectively accommodate cells, promote transmitter transmission, and new ECM formation (Feng et al., 2020).

### Hydrogel Material

Hydrogels can be prepared by physical crosslinking (through hydrophobic interaction, hydrogen bonding, electrostatic force, etc.) or chemical crosslinking (through covalent bonding). According to the source of hydrogels, it can be divided into three categories: natural, synthetic and composite hydrogels. Among them, natural materials include fibrin, alginate, chitosan, etc., which have great advantages in biocompatibility and low cytotoxicity, and thus are widely used in nucleus pulposus regeneration and repair. (Figure 1). However, its mismatched mechanical properties lead to the failure of the implant in the late stage of disk degeneration due to structural damage caused by long-term compression. Therefore, how to improve the mechanical properties of hydrogels is the focus of researchers. Gan et al. developed an interpenetrating network (IPN) reinforced and toughened hydrogel for NP regeneration, which has advantages such as NP-like mechanical properties and high toughness. The encapsulation of NP cells into the hydrogel clearly showed enhanced cell proliferation, natural cartilage formation phenotype, and ECM secretion. *In vivo* studies have also verified that IPN hydrogels can support cell retention and survival for a long time, thus promoting the rehydration and regeneration of degraded NP (Gan et al., 2017). Although the stiffness and toughness of the double network hydrogels were significantly improved, the long preparation time or continuous preparation made *in situ* curing difficult. The use of composite hydrogel is an alternative method, which has higher stiffness and strength, but also retains the characteristics of one-step hydrogel preparation, shortens the curing time, and is suitable for *in-situ* insertion. Schmocker et al. developed a composite hydrogel with functional properties like those of natural bovine NP. Disk height was restored from 65.6 to 99.0% in an *in vitro* study and remained the same after 500,000 loading cycles. Fifteen days after implantation, a continuous, undisturbed tissue/implant interface was observed histologically. This kind of composite hydrogel with excellent mechanical properties has great potential for clinical application (Schmocker et al., 2016).

**FIGURE 2:**
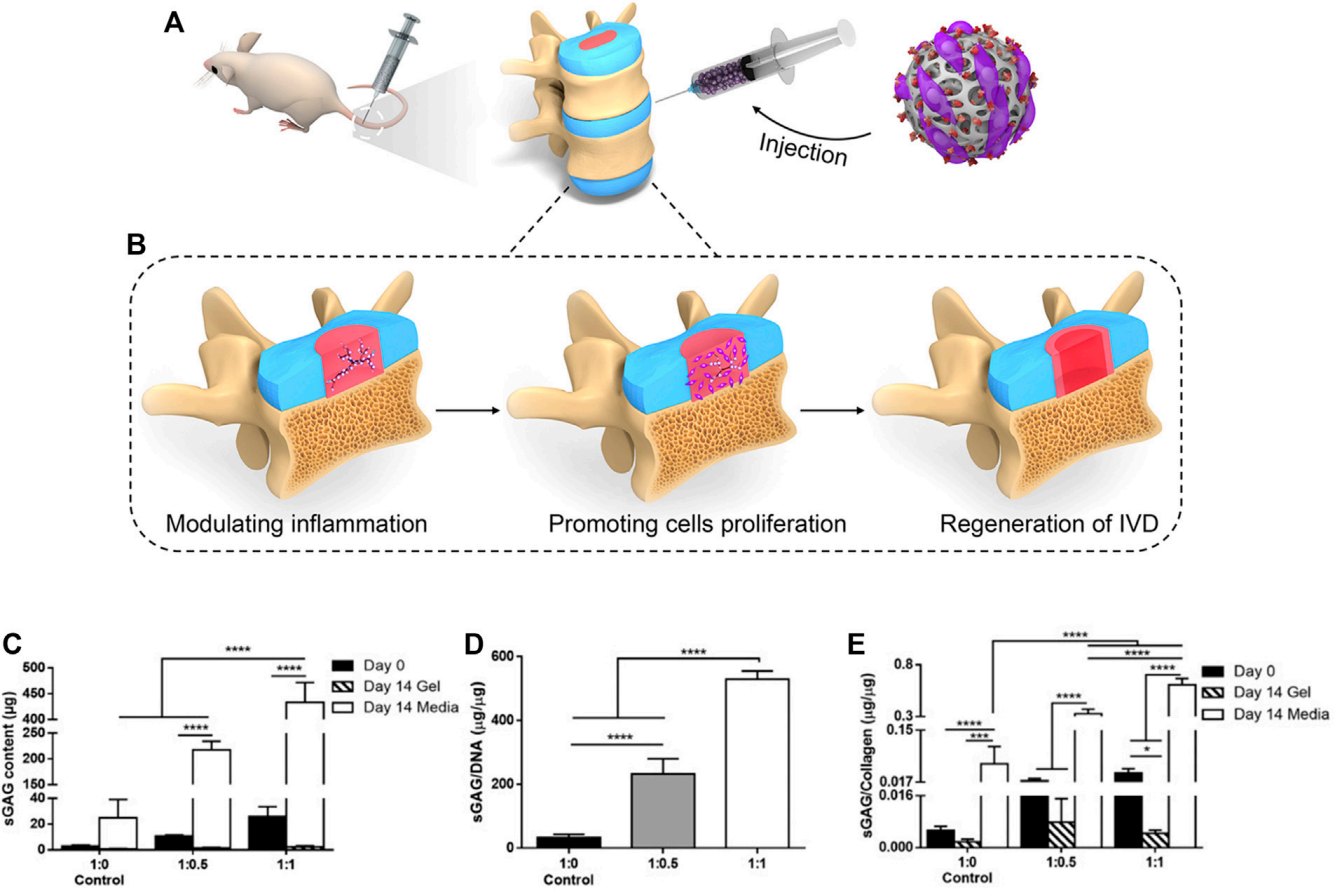
**(A)** The injection of Hydrogels in the rat model of IVD degeneration (**(A)** Adapted from Borrelli and Buckley (2020)) **(B)** The function provided by hydrogel in the regeneration of IVD degeneration, including modulating local inflammation, romoting nucleus pulposus cells proliferation, and regeneration of the intervertebral disk (IVD) **(C,D)** Synthesis of sGAG was found to be dependent on the initial gel composition, with significantly more sGAG being deposited by cells in gels containing higher initial sGAG. **(E)** The sGAG/collagen ratio was found to increase linearly with increasing CS present in the initial gel composition. (**(B–E)** Adapted from Bian et al. (2021)).

The degeneration of the nucleus pith is characterized by loss of hydration and tissue sclerosis. Histologically, decreased cell density, loss of sulfated glycosaminoglycans (sGAGs) and type II collagen, and an increase in type I collagen can be observed. In response to this, Chiara Borrelli et al. proposed that the production of sGAG could be increased by adding chondroitin sulfate to the hydrogel, and found that its presence is critical to the synthesis of type II collagen.(Figure 2) The phenotype of the notochord-like NP cell population changes to a more fibroblast-like state (Chen et al., 2009; Risbud et al., 2015). Previous studies have shown that laminin 111 functionalized soft polyethylene glycol (PEG) substrate can re-express the juvenile phenotype of degenerative nucleus pulpocytes, suggesting that soft substrate plays a role in phenotypic recovery of degenerative nucleus pulpocytes (Gilchrist et al., 2011; Francisco et al., 2014; Fearing et al., 2019). However, in the study of NP-derived stem cells (NP-SCs), Navaro et al. found that cell proliferation and differentiation were independent of matrix hardness in the study of NP-SCs but emphasized the influence of matrix modulus on the fate of NP-SCs (Navaro et al., 2015). In a recent study, Barcellona et al. confirmed this point. We developed a dipeptide-functionalized hydrogel scaffold with adjustable mechanical properties and adherent ligand presentation to control NP cell morphology and phenotype. In experiments, it was demonstrated that strict control of peptide selection and peptide presentation induces younger NP cell phenotypes regardless of substrate hardness, so it could be adhesion ligand presentation that really affects the phenotypes of nucleus pulpocytes (Barcellona et al., 2020).

Loss of nutrients from the stent limits its effectiveness in promoting disk regeneration. Studies have shown that NP cells need oxygen and other nutrients to synthesize matrix molecules, and different oxygen concentrations also affect the composition ratio of ECM (Mwale et al., 2011). Sun et al. used perfluorotributylamine (PFTBA) to regulate oxygen without affecting alginate. The survival and proliferation of human NP cells cultured in PFTBA-rich alginate scaffolds were enhanced, and the ECM was modulated in intervertebral disk tissue. Disk height and ECM were restored in a mouse model of intervertebral disk degeneration, showing beneficial effects in alleviating intervertebral disk degeneration (Sun et al., 2016).

### Gene Delivery Biomaterials

After NP degeneration, phenotypic changes of NP cells resulted in an imbalance of ECM anabolism and catabolism. Current treatment methods, such as symptomatic treatment or surgical treatment, can relieve symptoms in the short term but cannot solve the fundamental problem. Genetic modification of intervertebral disk cells through controlled and specific delivery of genetic material (DNA or RNA) is a promising therapeutic approach (Figure 1) (Roh et al., 2021). Many researchers have successfully transferred genes into intervertebral disk tissues through viral gene delivery systems (Figure 1) (Leckie et al., 2012; Han et al., 2021), but the obvious side effects limit further clinical application. The problem with retrovirus vectors is the risk of insertion mutation, while the problem with adenovirus vectors is the immunogenicity of transduced cells (Tripathy et al., 1996; Wallach et al., 2006), and several patients have even died after viral vector administration in clinical trials (Evans et al., 2008). From a safety viewpoint, nonviral vectors and poly micelles are better choices for intervertebral disk gene therapy (Figure 1). Zhang et al. developed a new type of hyperbranched polymer (HP) carrier to deliver anti-miR-199a (AMO), combined with biodegradable poly (lactic acid-co-glycolic acid) (PLGA) nanospheres (NS) to sustainably release AMO, and by blocking miR-199a, can upregulate hypoxia inducible factor (HIF)-1α. NF-SMS as a mesenchymal stem cell (MSC)-carrying scaffold can finally promote MSC NP differentiation and inhibit its osteogenic differentiation while enhancing NP tissue regeneration (Feng et al., 2020). Feng et al. also synthesized a mixed composite micelle (MPM) for the delivery of therapeutic plasmid DNA (pDNA), which was verified by *in vitro* and *in vivo* evaluations to improve the transfection efficiency of NP cells. After binding to heme oxygenase-1 (HO-1) pDNA, it can weaken the inflammatory response and increase the production of NP ECM, thereby slowing intervertebral disk degeneration (Feng et al., 2015).

## Biomaterials for Annulus Fibrosus Repair

In the process of low-back pain caused by intervertebral disk degeneration, NP degeneration certainly plays a leading role, but the incomplete AF structure is also one of the reasons for the occurrence and recurrence of low-back pain. According to research, most intervertebral disk degeneration is accompanied by AF damage, mostly after the NP herniation (Tavakoli et al., 2020). At this time, many of the curative effects currently targeted only at NP regeneration will be greatly reduced, such as cell therapy and bioactive factor therapy. Most of these therapies deliver drugs by local injection. After the AF is damaged, it will cause leakage of the injected material, which makes the injected material unable to stay in place for a long time and cannot exert its original effect. The structural integrity of the AF is the basis for the NP to maintain hydrostatic pressure. So, even if the NP is perfectly regenerated with AF unrepaired, it will cause the NP to herniate again under the action of axial pressure.

Therefore, scientists have long recognized the importance of AF regeneration. At the earliest, scientists used customized and personalized sutures to suture AF fractures or used polymer meshes and anchored them on the adjacent vertebral bones (Bailey et al., 2013; Vukas et al., 2015; Bowles and Setton, 2017). However, these two types have not been widely used due to the long operation time and high operation cost, and some studies believe that this treatment can only reduce the pain and the rate of re-herniation of the NP for a certain period but cannot restore the original structure and function of the AF and accelerate the degeneration risks of the intervertebral disk (Trummer et al., 2013; Choy et al., 2018).

### Hydrogel Material

**FIGURE 3:**
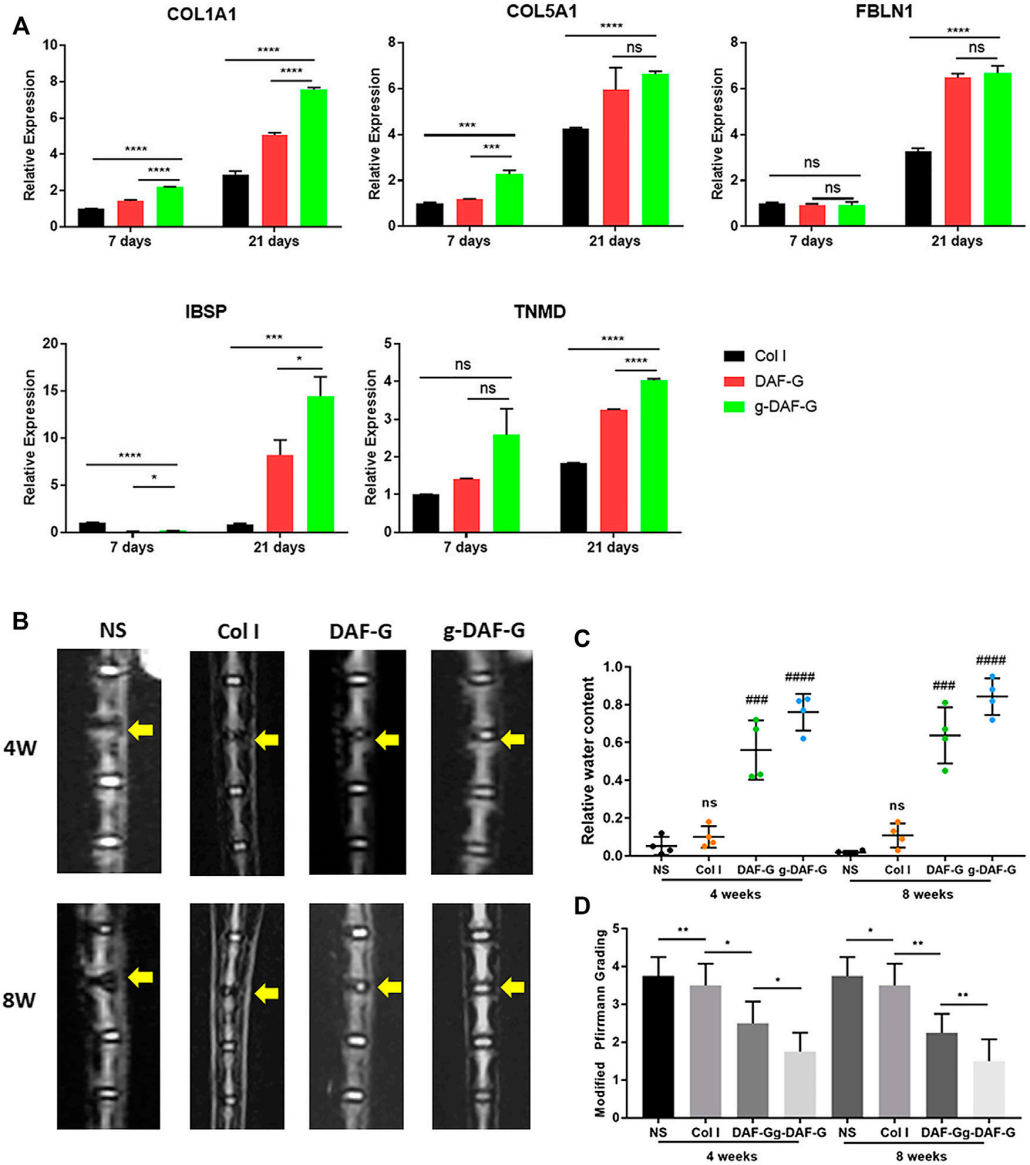
**(A)** The level of expression of AF-specific genes (collagen-1A1(COL1A1), collagen-5A1(COL5A1), fibulin-1 (FBLN1), integrin-binding sialoprotein (IBSP), and tenomodulin (TNMD)) was significantly higher in the DAF-G and g-DAF-G groups than Col I group on Day 21, which indicated the potential induction of directing specific differentiation of hBMSCs towards AF cells on DAF-G and g-DAF-G hydrogels. **(B)**The T2-weighted images showed that intervertebral space became narrower and the signal intensity of the NP region sharply declined in normal saline (NS) group as compared with the adjacent and intact disks. **(C)** Quantitative analysis showed that relative water content of DAF-G and g-DAF-G groups were larger than the Col I gel and the NS groups, indicating a potential regenerative function of AF-derived hydrogels on AF tissue defect. **(C,D)** The restorative effect analyzed by quantitative analysis and modified Pfirrmann grading was better in g-DAF-G than DAF-G group, revealing that the mechanical strength improved by genipin might be a vital factor in AF repair. (**(A**–**D)** Adapted from Peng et al. (2020a)).

Aiming at the idea of local repair, the biological patch applied to AF has been extensively studied. At present, several natural polymers or synthetic biological materials have been proven to effectively reconstruct the damaged structure of AF and prevent NP from hemiation again. For example, a hydrogel, a natural polymer, can be used for both NP and AF regenerations. Peng et al. developed an injectable genipin cross-linked acellular AF hydrogel (g-DAF-G) and proved that it has better biocompatibility, biological activity, and higher mechanical strength than a non-crosslinked acellular AF hydrogel, providing a simple and quick treatment alternative for repairing AF damage or tear (Figure 3) (Peng et al., 2020a). Another study found that, by adjusting the concentration of cross-linked genipin and changing the cross-linking conditions, its physical and chemical properties can be controlled to meet the different requirements for restoring the AF and NP tissue (Wang et al., 2020). There has also been a study on the optimal injection time of the hydrogel (Liu Z. et al., 2020). The study selected rhesus monkeys to construct a model of intervertebral disk degeneration, used MR to evaluate degeneration degree, and proved that the moderate degenerative stage (T1ρ value from 95 to 80 ms) may be the best time for hydrogel injection for regenerative intervention. Fibrin gel is also considered one of the most commonly used natural polymers in AF regeneration. Because AF tissue is rich in collagen, it has a lower immune rejection response. Cruz et al. studied the mechanical properties of genipin-cross-linked fibrin gels formed at different genipin concentrations (Cruz et al., 2017). The results showed that, as the genipin concentration increased, the compressive strength and shear modulus of the gel increased, surpassing the natural AF tissue. However, the improvement of the mechanical properties of fibrin gel will bring certain side effects, resulting in a decrease in the survival rate of local cells, because the gel is too hard and will hinder the supply of nutrients. Studies have found that 6% genipin can maintain a good cell survival rate and achieve sufficient mechanical strength. Another study found that high-density collagen gel (HDC) cross-linked with riboflavin can also be used for AF repair (Moriguchi et al., 2018). The study compared the disk height index (DHI), the size and the hydration of the NP of the untreated group, the cross-linked HDC group, and the cross-linked HDC group injected with AF cells after 5 weeks, and found that the average DHI of the two HDC gel groups exceeded that of the control group at 5 weeks. Compared with the cell-free HDC gel, the HDC gel loaded with AF cells had an acceleration repair the role of sealing. It has been proven that HDC gel loaded with AF cells has a better ability to repair ring defects after acupuncture than cell-free gel. Borem et al. proved that the addition of Interlamellar matrix (ILM) GAG can also effectively improve the ability of the patch to resist impact loads and can prevent natural IVD tissues from herniating during the application of super-physiological load (5.28 ± 1.24 MPa), which is a repair strategy that can be used for the AF of focal ring defects (Borem et al., 2019). In addition, many biosynthetic materials also show certain advantages in AF repair, such as D, l-acryloyl ester and trimethyl carbonate can have morphological memory function, poly (trimethylene carbonate) (PTMC) combined with elastic PU film can prevent bovine NP from protruding again within 14 days under dynamic load, and PCL triol malate can be degraded, so that the mechanical properties of the fiber ring can be adjusted (Pirvu et al., 2015).

In recent years, the research on hydrogels for the repair of fibrous annulus has become more and more in-depth. Peng et al. used bovine fibrous annulus acellular tissue matrix to make a hydrogel, and the integrin-mediated RhoA/LATS/YAP1 signaling pathway can promote the specific differentiation of stem cells into AF for AF repair (Peng et al., 2021). In addition, Liu et al. used the bionic structure to prepare the anisotropic wood cellulose hydrogel, which is mainly responsible for stress buffering and energy absorption (at least ∼60% energy dissipation) just like the AF tissue (Liu et al., 2021). However, due to poor adhesion or low failure strength between hydrogels and wet tissue surface, DiStefano et al. developed a double-modified glycosaminoglycan to covalently bond hydrogels to AF tissue, thus optimizing the sealing method of AF defect (DiStefano et al., 2020).

### Nanofiber Material

**FIGURE 4:**
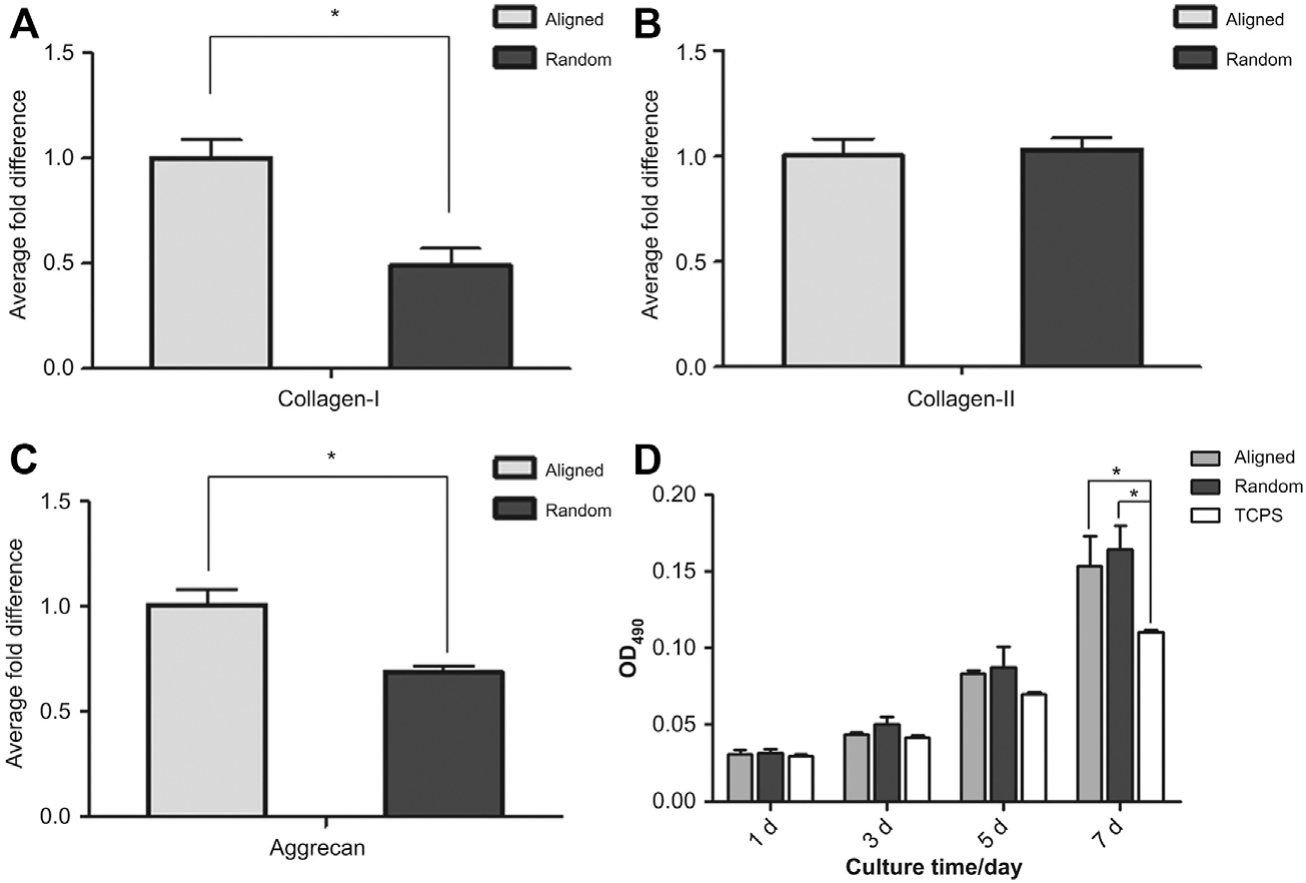
**(A)** Real-time quantitative PCR was performed to compare the expression of collagen-I, collagen-II and aggrecan genes in AFSCs cultured on aligned and random scaffolds. After 7 days of culture, the expression level of collagen-I in AFSCs cultured on aligned scaffolds was almost twice that of cells grown on random scaffolds. **(B)** The expression of collagen-II was similar in both AFSCs cultured on aligned and random scaffolds. **(C)** The expression pattern of aggrecan was similar to that of collagen-I; cells cultured on aligned scaffolds had approximately 0.4 times higher aggrecan gene expression than those grown on random scaffolds. **(D)** There was no apparent difference in cell proliferation between AFSCs cultured on aligned scaffolds and those on random scaffolds. (**(A**–**D)** Adapted from Liu et al. (2015)).

Some studies believe that both biological patches and suture technology only strengthen the AF but do not treat the inflammation that occurs in the AF (Wang et al., 2021). In this study, a PLGA/polycaprolactone (PCL) Zdextran (DEX) composite film loaded with testicular plastid extract (PTE) was prepared by electrospinning through an *in vitro* inflammation model. The cytocompatibility and anti-inflammatory effect of the material were verified, and we found that P10P8D2 (PLGA 10 g, PCL 8 g, DEX 2 g) composite nanofiber membranes exhibited the most uniform diameter distribution, best mechanical properties, moderate degradation rate, and best cell compatibility characteristics. It can simultaneously exhibit anti-inflammatory and cell proliferation promoting effects. Another studies have found that fibrosus poly (ether carbonate carbamide) urea (PECUU) scaffolds can regulate the differentiation of amniotic fluid-derived stem cells (AFSCs) attached to scaffolds by adjusting the changes in fiber size and elasticity of scaffolds (Zhu et al., 2016; Chu et al., 2021). On a scaffold with large fibers and strong elasticity, the expression of phenotypic marker genes in the outer annulus is upregulated; on a scaffold with small fibers and low elasticity, the expression of phenotypic marker genes in the inner annulus is enhanced. It has been proven that this is controlled by activating the CAV1-YAP mechanical transduction axis. Another study reached a similar conclusion. They proved that YAP-related proteins play a key role in influencing the differentiation direction of AFSCs (Chu et al., 2019). Liu et al. also contributed to the directional differentiation of AFSCs (Figure 4) (Liu et al., 2015). They proved that, compared with random stents, aligned fiber PU scaffolds can make AFSCs elongated and better aligned and increase the expression of collagen I and the proteoglycan matrix, providing a favorable microenvironment for the cells of the outer layer of AF cells. In summary, such a scaffold can effectively induce changes in cell shape, adhesion, and expression of ECM by adjusting fiber size so that engineered AF tissue has a hierarchical structure close to that of natural AF tissue (Zhou et al., 2021). Chen Liu et al. further improved the PECUU nanofiber scaffold (Liu Y. et al., 2020). They found that PECUU is compatible with the mechanical properties of the natural AF ECM but lacks the biological activity of the natural ECM. The decellularized AF matrix (DAFM) has good biocompatibility and biodegradability and has been proven to promote the secretion of AF-related ECM. It uses coaxial electrospinning technology to manufacture a DAFM/PECUU hybrid electrospinning stand. It was also proved that the gene expression and ECM secretion of type I and II collagens and proteoglycan of the annulus-derived stem cells cultured on the DAFM/PECUU electrospun scaffold exceeded that of the PECUU fiber scaffold. In another study, a new type of scaffold made of PCL was manufactured by 3D printing technology (Christiani et al., 2019). This new type of scaffold can also effectively simulate the structure and biomechanical properties of natural tissues. Annulus fibroblasts can attach and diffuse on it, and type I collagen, aggrecan, and aponeurotic protein, a protein marker specific to AF, can be effectively expressed. Cell sheet rolling system (CSRS) has also been used in manufacturing AF. Shamsah et al., 2019 used CSRS technology to make an electrospun fiber scaffold comprising a synthetic biopolymer mixture of PCL and poly (L-lactic acid) acid (PLLA), which realizes the anatomical structure of the AF with a complete 3D circular structure for the first time, and proved that bovine annulus cells can maintain vitality on it (Shamsah et al., 2019).

### Biomaterials for Cell Therapy

A major advantage of biomaterials is that they can be used to deliver cells. Cell therapy is a promising therapy that can be divided into endogenous and exogenous cell therapies (Figure 1). Endogenous cell therapy refers to local injection of cells to produce biologically active factors or direct injection of biologically active factors to stimulate *in situ* cell regeneration, while exogenous cell therapy regenerates AF by directly injecting annulus fibroblasts or stem cells that can be induced to differentiate into annulus fibroblasts. Because AF is a cell-deficient structure, coupled with the local inflammation caused by intervertebral disk degeneration, the number of AF cells *in situ* is further reduced. When stimulated by biologically active factors, it is not enough to proliferate and differentiate to compensate for the local inflammation of AF. This is the most severe challenge of endogenous cell therapy. The most important limitation of exogenous cell therapy is the poor retention of cells at the injection site. This is caused by the loss of anchorage, called anoikis, which can cause cell death (Discher et al., 2009).

Mesenchymal stem cell therapy is currently the most studied type of exogenous cell therapy (Sakai and Andersson, 2015). Some studies have reported that the injection of mesenchymal stem cells aggravates the degree of intervertebral disk degeneration (Vadala et al., 2012). This is because the cells injected in this study leaked. In most studies that did not leak, bone marrow mesenchymal stem cells (BMSCs) can effectively differentiate into intervertebral disk cells, promote the synthesis of intervertebral disk ECM (Chiang et al., 2019), and improve the clinical outcome of patients with intervertebral disk degeneration (Sun et al., 2019). Therefore, many scholars have recognized the idea that mesenchymal stem cells can regenerate intervertebral disks. At present, many researchers have focused on how to improve the delivery method of cells, how to induce the directional differentiation of mesenchymal stem cells, and how the delivered cells can maintain their biological activity for a longer time (Tibiletti et al., 2014). After years of exploration, researchers have discovered that biomaterials are effective carriers for cell therapy (From the American Association of Neurological Surgeons et al., 2018). More and more engineering materials, including natural polymers, synthetic polymers, and their combinations, have been applied to intervertebral disk repair (Peng et al., 2020b). This is because biological materials can isolate these cells used for intervertebral disk regeneration from the inflammatory environment or other factors that cause the harsh biological environment of the intervertebral disk and can keep these cells at the injection site (Bertram et al., 2005). Therefore, how different materials affect stem cells is the focus of current research. Compared with cell therapy for NP regeneration, there are relatively few researchers on cell regeneration therapy for the AF. This is also related to the high complexity of the AF structure and the higher requirements for balancing mechanical and biological properties. Bhunia et al. created a multi-layer disk-shaped stratum corneum structure based on silk protein that comprises concentric lamellar layers, which mimics the native hierarchical structure of AF, and can effectively support cells’ proliferation, differentiation, and deposition of ECM, which can promote AF regeneration (Bhunia et al., 2018). Adding to the structure of the material, the biologically active factors added to the material also play an important role in the efficacy of cell therapy. Blanquer et al. found that adding transforming growth factor (TGF)-β3 to an AF scaffold constructed with PTMC by stereolithography can support the differentiation of human adipose stem cells into AF (Blanquer et al., 2017). Fibrin gel inoculation can better the distribution and proliferation of adipose stem cells and the production of AF-like matrix. Not only is the production of sulfated sGAG and collagen significantly upregulated but the formed collagen is also oriented and arranged into bundles in the designed pores. Although natural polymers generally have better biocompatibility and better similarity to natural microstructures and ingredients, their mechanical properties are often limited. Therefore, a synthetic matrix with a good composition and controllable biodegradability is studied. Pirvu et al. developed a PTMC scaffold covered with a PU membrane and planted with human BMSCs, which can withstand dynamic loads, restore the fractured bovine AF tissue of the height of the intervertebral disk, prevent NP protrusion, and differentiate *in situ* (Pirvu et al., 2015).

Although exogenous cell therapy has achieved good results in AF regeneration, these cell carriers made of natural polymers or synthetic polymers remain foreign bodies compared to the human body, which can cause more or less immune rejection. Concurrently, the process of delivering these materials to the degenerated intervertebral disk of the human body is an invasive operation that will cause secondary damage to the AF and NP. Therefore, endogenous cell therapy based on the concept of inducing *in situ* cell regeneration and proliferation should be the main direction of future researchers’ efforts. At present, researchers have found that using the homing effect of stem cells can effectively overcome the deficiencies of endogenous cell therapy (From the American Association of Neurological Surgeons et al., 2018). BMSCs homing is the process of recruiting cells from the initial biological environment to damaged or pathological tissues. When stem cells are stimulated by a certain biologically active factor, they are mobilized into the peripheral bloodstream and migrate to damaged tissues or organs (Fong et al., 2011). However, the current understanding of the exact process and mechanism of how BMSCs are mobilized and guided to the position of the effector remains incomplete. However, researchers believe that BMSCs are jointly guided by multiple cell signaling molecules, and the damaged tissue itself expresses unique receptors or ligands to promote the penetration of BMSCs into the affected area (Sordi, 2009). At present, it has been proven that there are various growth factors and chemokines that can induce the migration of mesenchymal stem cells, such as tumor necrosis factor-α, interleukin-1β (IL-1β) (Ponte et al., 2007), insulin-like growth factor-1 (IGF-1), and platelet-derived growth factor-AB (PDGF-AB), etc., but these have an inducing-migration effect on various stem cells, and researchers have found that the chemokine that has the greatest impact on BMSCs is RANTES (also known as CCL5), Macrophage-derived chemokine (MDC), and stromal cell-derived factor-1 (SDF-1), and these are all elevated in degenerative disk tissues (Pattappa et al., 2014). However, it is frustrating that studies by Tam et al. and Cunha et al. found that the ability of stem cells to enter the body through intravenous injection is relatively limited (Tam et al., 2014; Cunha et al., 2017). This may be related to the lack of blood vessels in the intervertebral disk, but, in human degenerated intervertebral disks, there are many new blood vessels that grow in, which may not be simulated by animal experiments, and these new blood vessels may potentially promote BMSCs, reaching the damaged area and promoting regeneration.

## Biomaterials for Cartilage Endplate Repair

**FIGURE 5:**
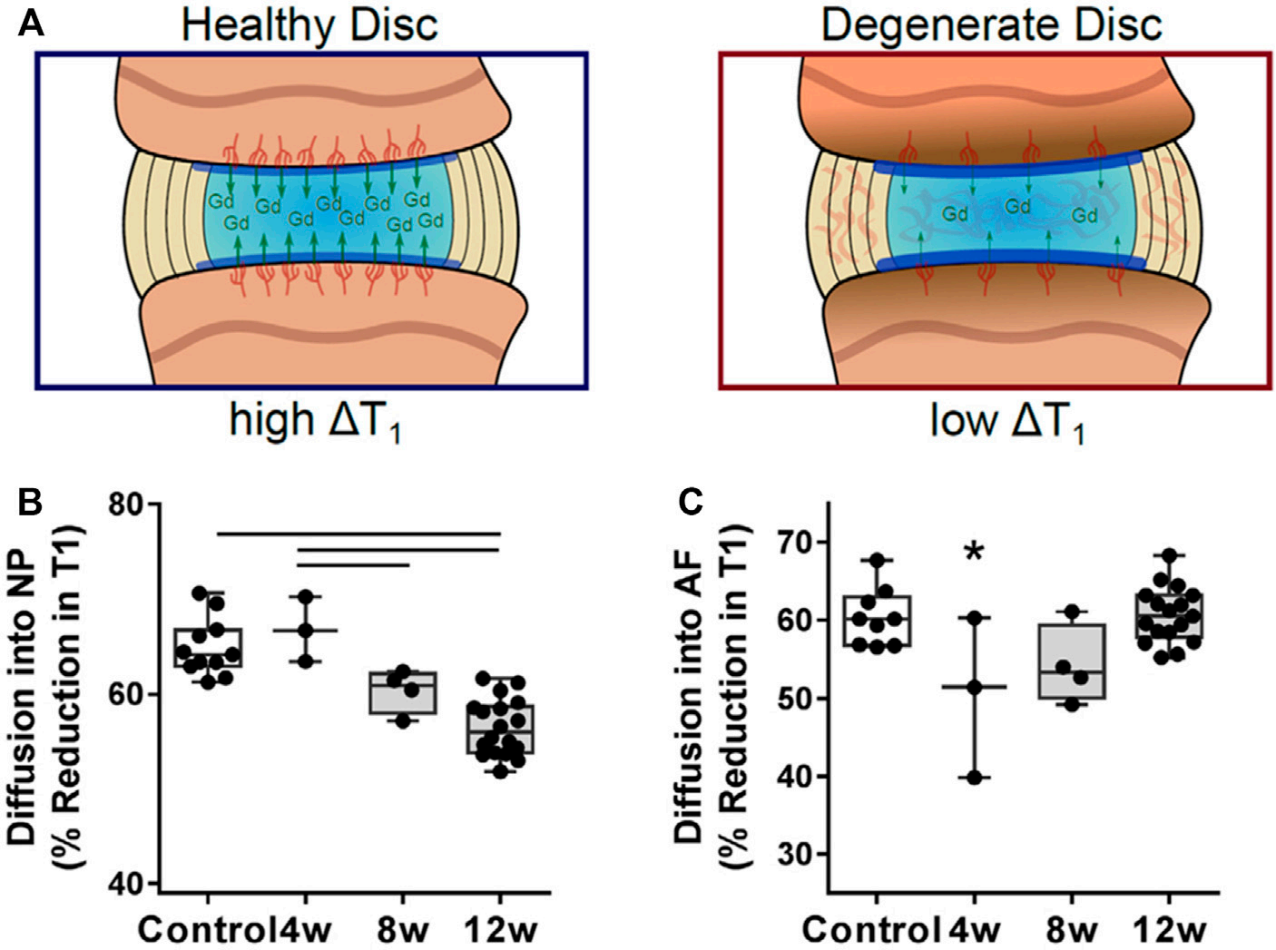
**(A)** The change in T1 relaxation time within the intervertebral disk after administration of the contrast agent gadodiamide **(B)** Within the NP, gadodiamide diffusion into the disk progressively decreased and was significantly lower at 12 weeks compared with healthy controls. **(C)** Diffusion into the AF was significantly reduced at 4 weeks post-puncture, followed by a return to control levels. (**(A**–**C)** Adapted from Ashinsky et al. (2020)).

Endplate is the main way for metabolites to enter and exit intervertebral disk (Malandrino et al., 2014), and an insufficient supply of essential nutrients and waste accumulation are often considered important factors inducing intervertebral disk degeneration (Figure 5) (Ashinsky et al., 2020). Calcification of endplate cartilage and occluded nutrient canals increase with age (Bernick and Cailliet, 1982). If the endplate is hardened or thickened, biomaterials implanted for disk repair may not have the desired effect. Therefore, biomaterials that restore the structure and function of the disk may not be sufficient, and the patency of the endplate requires consideration. In earlier studies, disk repair materials generally focused only on maintaining the structural and mechanical properties of the endplate. Ishiguro et al. developed a scaffold-free tissue-engineered construct (TEC), a 3D matrix comprising high density undifferentiated MSC. Intervertebral disk height, endplate, and AF were maintained 6 months after implantation in a rat model of disk degeneration, and age-related biomechanical impairment was reduced (Ishiguro et al., 2019). With the development of research, the importance of interface integration with cartilage endplates during disk repair has been greatly considered. Hamilton et al., *in vitro*, formed a three-phase construct comprising NP, CEP, and a calcium polyphosphate (CPP) matrix (as a bone substitute). After 8 weeks of culture, a continuous layer of NP tissue was formed and fused with the underlying cartilage tissue, which, in turn, was integrated with porous CPP to improve the interface properties of bone replacement and IVD tissue (Hamilton et al., 2006). In a recent study, Chong et al. attempted to construct a model of AF-cartilage interface *in vitro*, using contact co-culture from outer AF cells inoculated with an angular layer, multilayer PC PU scaffold, and articular chondral cells. After a few weeks, the OAF tissue was integrated with the cartilage and like the natural interface. The apparent tensile strength of the *in vitro* interface was also significantly increased, and it is expected to achieve clinical overall disk replacement by adding cartilage endplate repair (Chong et al., 2020). Currently, there are few studies on the repair and regeneration of cartilage endplates among intervertebral disk repair materials, but the importance of cartilage endplates in intervertebral disk repair has been discovered by more and more researchers. It is believed that there will be more studies on cartilage endplate repair in the future.

## Biomaterials for Intervertebral Disk Repair Using Combination Strategies

Intervertebral disk degeneration is a process in which the cartilage endplate, NP, AF, or biological environment co-occur with pathophysiological changes. The upper and lower facet joints and surrounding muscles can even undergo degeneration. Therefore, study on intervertebral disk regeneration should not only consider repairing one of them but should be extensively repaired to completely eradicate the low-back pain symptom. The above-mentioned cell therapy and tissue engineering methods have only discussed the impact on a single structure in various studies, but, in fact, their impact on the intervertebral disk is all-round—it is just that the impact on other structures is not significant or has no clinical significance for the time being. Therefore, it is imperative to find a way to regenerate the intervertebral disk in an all-round way or to organically integrate the above-mentioned structural regeneration methods.

Eliminating the inflammatory environment and restoring the original ECM state are considered effective methods to improve the intervertebral disk in an all-round way (Hofmann et al., 2018). To achieve this goal, the degenerated intervertebral disk must be transformed into a physiological state. Potential strategies include eliminating pro-inflammatory cytokines and proteases in the ECM; reversing the gene expression of pro-inflammatory cytokines and proteases in the resident intervertebral disk cells; and stimulating the resident intervertebral disk cells to produce new ECM. Based on this strategy, Guo found that various growth factors can regulate ECM synthesis, including the inhibition of inflammation and downregulation of degrading enzymes (Guo et al., 2021). However, growth factors, including TGF-β, fibroblast growth factor (FGF), and IGF-1 may induce unwanted blood vessel growth, thereby accelerating the process of intervertebral disk degeneration. Only GDF-5 can effectively alleviate intervertebral disk degeneration without inducing the ingrowth of blood vessels. Saberi et al. found that mitochondrial dysfunction is also one of the factors in intervertebral disk degeneration (Saberi et al., 2021). This is because mitochondria are the main source of cellular energy supply and the main source of reactive oxygen species, which is the main cause of the inflammatory environment of the intervertebral disk. First, it has been proven that MitoQ, MitoTEMPO, SkQs, XJB-5-131, and other regulating mitochondrial functions can effectively play the role of intervertebral disk regeneration. Bai et al. also reached a similar conclusion. They found that hydrogel scaffold-carrying rapamycin can effectively eliminate the active oxygen components in the microenvironment of the intervertebral disk, thereby playing a role in intervertebral disk regeneration (Bai et al., 2020). Another study found that acid-sensitive ion channels (ASICs), as key receptors for extracellular protons of central and peripheral neurons, are related to intervertebral disk degeneration and a decrease in microenvironmental pH. By downregulating the expression of ASIC2 and ASIC4 in human IVD, it can slow down the senescence of intervertebral disk cells. Several other studies found that CCL25 (Stich et al., 2018), colony-stimulating factor (Abdel Fattah and Nasr El-Din, 2021), anti-inflammatory drug etanercept (Li et al., 2020), chitosan/poly-γ-glutamate nanocomplex (Cunha et al., 2020), HIF (Kim et al., 2021), stromal cell-derived factor-1α (Zhang et al., 2018) and containing platelet plasma (Chang et al., 2020; Fiani et al., 2021) can play the role of intervertebral disk regeneration by inhibiting the local inflammatory environment. As mentioned earlier, some cells can also stimulate the proliferation of endogenous stem cells or progenitor cells by secreting a broad spectrum of biologically active factors to produce new ECM. Sun et al. found that connective tissue growth factor (CTGF) and TGF-β3 can effectively induce the corresponding BMSCs to differentiate into NP-like cells and fibroblast-like cells and rebuild the ECM (Sun et al., 2021). In addition, more and more experimental results show that stem cell-derived exosomes have potential regenerative capabilities by promoting cell proliferation, enhancing angiogenesis, promoting the restoration of ECM homeostasis, inhibiting inflammation and other unknown effects (Hingert et al., 2020; Hu et al., 2020). Some of these beneficial mechanisms can also be implemented in repairing intervertebral disk degeneration.

In addition, replacing the entire intervertebral disk is considered one of the potential methods for regenerating the entire intervertebral disk. In fact, a long time ago, researchers have studied the clinical effect of artificially synthesized intervertebral disks directly replacing severely degenerated intervertebral disks, but the results obtained are negative because this synthetic intervertebral disk only considers the mechanical properties of the intervertebral disk without considering the biocompatibility—this product is slowly being phased out. However, in recent years, a heterogeneous decellularized scaffold has attracted the attention of researchers (Hensley et al., 2018). Since this type of scaffold is derived from the body of cattle, worrying about the shortage of donors is needless. Due to the acellular treatment, it lacks immunogenicity and has achieved good results in many animal experiments (Fiordalisi et al., 2020; Norbertczak et al., 2020). However, the mammals used in these experiments are all four-legged, and the spinal column is parallel to the ground, so the load on their disks is not comparable to that in humans. So far, no corresponding clinical studies have proven that the acellular scaffold can effectively replace the function of the natural disk in humans.

Although most of the above regeneration strategies have achieved positive results, most of the treatment methods can only be applied to the early stages of intervertebral disk degeneration because most studies have proven that, whether it is stem cell therapy or growth factor therapy, for late degeneration, the harsh local environment of the intervertebral disk will cause the rapid loss of the biological activity of the injected substance, and then will cause these effective treatments to fail to achieve their intended effects. Therefore, the concept of intervertebral disk degeneration should be considered. It should be regarded as a disease like diabetes and hypertension. Tertiary prevention should be initiated, starting with primary prevention, such as the cause of the disease, and strengthening publicity and education to inform people of the seriousness of the disease and start early prevention. However, now, because the early degeneration of the intervertebral disk is symptomless and difficult to find, the current basic research requires an imperative development to find the biomarkers of early intervertebral disk degeneration and to intervene in time while avoiding the widespread occurrence of low-back pain in the world.

Although we advocate early intervention, the early symptoms are mild, and most of the current methods of administration are local injections. This is an invasive operation that will damage the surrounding muscles and joint capsule. If you want to inject the NP, it will definitely damage the AF. The damage to these tissues all has the risk of aggravating intervertebral disk degeneration, and whether the therapeutic effect brought by local injection of drugs can offset the risk of this damage remains unexplored. Therefore, finding a better drug delivery method with less trauma is imperative.

## Preclinical Study of Biomaterials for Intervertebral Disk Repair

In preclinical studies of intervertebral disk repair, rats are the most common animal model. Barcellona et al. prepared a peptide functionalized hydrogel for the study of intervertebral disk repair (Barcellona et al., 2021). The gel has a stiffness similar to that of the degenerated nucleus pulposus (∼10 kPa), which is conducive to simulating the cell living environment and playing a certain mechanical support role. After functionalization with laminin-mimetic peptides IKVAV and AG73, it can provide the delivered cells with biomimetic cues in order to promote phenotypic expression and increase biosynthetic activity. In the rat intervertebral disk degeneration model, it was also confirmed that after the gel was combined with primary NP cells, the cell retention rate and survival time were improved, and the degeneration was significantly improved. In the process of intervertebral disk repair, excessive local inflammatory reaction will greatly hinder the regeneration of intervertebral disk tissue. Therefore, in order to make the repair of the intervertebral disk proceed smoothly, many researchers have also tried *in vivo* experiments to suppress the inflammatory reaction that occurs during the degeneration. Bian et al. developed a hydrogel microsphere with an elastic modulus of 25.23 ± 2.58 kPa, which is sufficiently rigid to protect the cells from shear force during injection and avoid other mechanical stresses during application. Subsequently, the hydrogel microspheres loaded with NP cells and pro-inflammatory factor expression inhibitors were injected into the degenerated intervertebral disks of rats. It was observed that the reduction of inflammatory factors and the increase in the proliferation activity of NP-loaded cells and the increase in ECM deposition were all helpful to the regeneration of the intervertebral disk (Bian et al., 2021). While the degeneration of the intervertebral disk produces inflammation, the most urgent problem to be solved is pain. Mohd Isa et al. developed a new rat model of pain caused by intervertebral disk injury and proved that hyaluronic acid hydrogel can reduce inflammation while reducing pain, providing a method for the treatment of back pain caused by intervertebral disk degeneration (Mohd Isa et al., 2018).

The current research of biomaterials is still mainly based on *in vivo* experiments in small animals. The reason is that it is difficult for materials to achieve sufficient mechanical strength while ensuring biological activity, while *in vivo* researches on large animals are mainly conducted on artificial intervertebral discs, which pay more attention to their long-term mechanical properties and durability. Shikinami et al. developed a flexible artificial intervertebral disc system. Compared with the traditional rigid system composed of a solid plate and a core material, it has been verified in baboons for its long-term durability and biocompatibility. At the same time, it simulates the movement of human intervertebral discs. The mechanical test also proved that the durability can reach a normal person for more than 50 years (Shikinami et al., 2010). The current clinical research on the alternative treatment of intervertebral disc degeneration is mainly total disc replacement, and different artificial disc materials have a certain impact on the clinical efficacy (Coban et al., 2021). At the same time, the high recurrence rate and unclear risk factors for secondary surgery also limit the development of surgery (Perfetti et al., 2021). The clinical transformation of artificial intervertebral discs still needs more in-depth and long-term research.

## Concluding Remarks and Future Perspectives

We review the current approaches to biomaterial-based repair of degenerative intervertebral disks, including disk replacement, cell therapy, and gene therapy. Tables 1–3 summarizes the biomaterials used in various intervertebral disk repair methods and their repair functions. Early studies focused on the restoration of the intervertebral disk structure and ignored the interaction between cells, so the success rate of intervertebral disk repair was not high. In a later study, many researchers began to pay close attention to the use of cell therapy to weaken and repair intervertebral disk degeneration, mainly because most of the cells are mesenchymal stem cells or between intervertebral disk cells. Through biomimetic ECM or form to ECM formation environment, promote cell proliferation and differentiation, thus to achieve the effect of repair of intervertebral disk. Although gene therapy uses viral vectors to treat degenerative disk disease and has achieved certain effects, the main problems of this method include carcinogenesis and immune response. Therefore, non-viral gene vectors are the preferred delivery system, although the transfection efficiency is lower than that of viral vectors. Intervertebral disk degeneration restorative materials mainly include water gel, nanofiber, etc., through the combination can be simulated natural intervertebral disk tissue regeneration of NP in the study, usually has a similar to natural NP material performance elastomer, and in the AF regeneration repair, is more concerned with the toughness of the material, the purpose is to form good sealing effect and prevent NP herniation again. Concurrently, many studies have been conducted to repair the cartilaginous endplate. Because the endplate affects the nutritional and metabolic exchange of the intervertebral disk after the material is implanted, the interface integration of the endplate with the NP and the AF is also important. The specific method is to form the cartilage layer through the chondrogenic differentiation ability of the material, and then fuse with the NP or AF to achieve interface contact. Otherwise, the accumulation of metabolic waste and nutrient deficiency will induce the failure of the repair process. In fact, disk degeneration is not a single degeneration, but the biological environment of the NP, AF or cartilaginous endplate has changed. Therefore, the repair method should be multi-faceted to eradicate the symptoms completely. Currently, biomaterials for repairing intervertebral disks have begun to have multifaceted repair functions, and this combined repair strategy is expected to achieve the overall repair of intervertebral disks. However, notably, research on intervertebral disk regeneration materials still faces many challenges. Requirements for materials with mechanical properties parallel natural intervertebral disk tissue, and after reaching long-term exercise load, they can still maintain a high standard of their structure and function. Concurrently, the materials also need to have the ability to integrate cartilage endplates to achieve the smooth exchange of nutrition and metabolism. In addition, pathophysiologically, circadian rhythm disorders, intervertebral disk cell apoptosis and senescence. Problems such as abnormal autophagy of the inflammatory response also provide great challenges for disk repair with biomaterial. Therefore, to achieve true disk regeneration and repair, both the improvement and development of biomaterial alongside a deeper understanding and study of the pathophysiology of disk degeneration are needed.

**TABLE 1:** Summary of various biomaterials for nucleus pulposus repair and their functions.

Material type	Material composition	Target cell/gene		*In vivo* experimental model	Repair effect	References
Nanofiber	PC-PU		Bovine NPCs and human MSCs	Bovine whole IVD organ culture model	Restore the mechanical properties of enucleated disks and delay further degradation of natural disk tissue	Li et al. (2016)
	PLLA NF-SMS		Rabbit MSCs	Rabbit lumbar degeneration model	Help mesenchymal stem cell adhesion, proliferation and np-like differentiation, promote transmitter transmission and new ECM formation	Feng et al. (2020)
Hydrogel	Dextran, gelatin and poly (ethylene glycol)		Porcine and rat NPCs	Rat tail IVD and porcine disk degeneration model	Support long-term cell retention and survival in the rat IVDs, and facilitate rehydration and regeneration of porcine degenerative NPs	Gan et al. (2017)
	Poly (ethylene glycol) dimethacrylate		NA	Bovine disk model	Has good mechanical properties and tissue integration ability, and maintain the height of the disk	Schmocker et al. (2016)
	Adducts of fibrinogen and poloxameric block copolymers called Tetronic 1307		Porcine NP–SCs	NA	Cell proliferation and differentiation have nothing to do with matrix hardness, but matrix modulus can affect the fate of NP-SCs	Navaro et al., 2015
	Poly (ethylene glycol), IKVAV and AG73 peptides		Human NP cells	NA	Close control of peptide selection and peptide presentation can lead to a more juvenile NP cell phenotype regardless of substrate stiffness	Barcellona et al. (2020)
	PFTBA and alginate		Human NP cells	Mouse disk degeneration model	Promote NP cell survival and proliferation, restore the disk height and the ECM, indicate its beneficial effect on alleviating disk degeneration	Sun et al. (2016)
Gene therapy	Hyperbranched polymer and AMO		Rabbit MSCs/MiR-199a	Rabbit lumbar degeneration model	Promote MSC nucleus pulposus differentiation and inhibit its osteogenic differentiation, and enhance nucleus pulposus tissue regeneration	Feng et al. (2020)
	MPM and HO-1 pDNA		Rabbit NP cells/HO-1 pDNA	Rat tail disks	Weaken the inflammatory response and increase the production of NP ECM, slow the degeneration of the intervertebral disk	Feng et al. (2015)

**TABLE 2:** Summary of various biomaterials for AF repair and their functions.

Material type	Material composition	Target cell/gene		*In vivo* experimental model	Repair effect	References
Surgical suture	Anulex Technologies, Minnetonka, MN		NA	Patients with lumbar disc herniation	Reduce the need for subsequent reherniation surgery while retaining the benefits of discectomy with no increased risk for patients	Bailey et al. (2013)
	an anular closure device		NA	Patients with lumbar disc herniation	Provide very low rates of disk reherniation and exhibit excellent disk height maintenance and sustain disability, leg pain, and back pain improvement within a 24 months postoperative study period	Vukas et al. (2015)
Hydrogel	g-DAF-G		Human BMSCs	Rat tail acupuncture degenerative model	Exhibit well biocompatibility, great bioactivity, and much higher mechanical strength	Peng et al. (2020a)
	Genipin cross-linked fibrin gel (FibGen) adding PDLGA		NA	NA	FibGen is highly modifiable with tunable mechanical properties that can be formulated to be compatible with human AF compressive and shear properties and gelation kinetics and injection techniques compatible with clinical discectomy procedures	Cruz et al. (2017)
	HDC cross-linked with riboflavin		AF cell	Needle puncture-induced degeneration rat model	AF cell-laden HDC gels have the ability of better repairing annular defects than acellular gels after needle puncture	Moriguchi et al. (2018)
	ILM-GAG		NA	NA	Adding ILM glycosaminoglycan (GAG) can enable the annulus repair patch (AFRP) to withstand stronger impact strength and prevent the nucleus pulposus from herniating again when over physiological load	Borem et al. (2019)
	AF decellularized tissue matrix		Human BMSCs	AF defect rat model	AF specific differentiation of stem cells and AF tissue regeneration	Peng et al. (2021)
	Anisotropic wood cellulose hydrogel		Mouse BMSCs	NA	Possesses imitation and construction of the natural structure, favorable mechanical matching and good biocompatibility and unique mechanical buckling buffer characteristics	Liu et al. (2021)
	Double-modified glycosaminoglycan		NA	*Ex vivo* bovine model of discectomy	Covalently bond hydrogels to AF tissue to seal AF defect	DiStefano et al. (2020)
Nanofiber	PLGA/PCL DEX		AF cells	*in vitro* inflammation model induced by interleukin (IL)-1β	The nanofiber membrane composed of P10P8D2 and PTE has anti-inflammatory and pro-proliferation effects on AF cells	Wang et al. (2021)
	PECUU		AF-derived stem cells	NA	YAP-related proteins play a key role in influencing the differentiation direction of AFSC. The scaffold can effectively induce changes in cell shape, adhesion and expression of ECM by adjusting the fiber size, so that the engineered AF tissue has a layered structure close to that of natural AF tissue	Chu et al. (2019)
	DAFM/PECUU-blended electrospun scaffolds		AF-derived stem cells	NA	Gene expression and ECM secretion of collagen type I and II and aggrecan from AF-derived stem cells cultured on DAFM/PECUU electrospun scaffolds were higher than from those on PECUU fibrous scaffolds	Liu et al. (2020)
	PCL		Bovine AF cell	NA	Bovine AF cells can be attached and diffused on it, and type I collagen, aggrecan, and AF specific protein markers can be effectively expressed	Christiani et al. (2019)
	PCL and PLLA		Bovine AF cell	NA	For the first time, a custom-built Cell Sheet Rolling System (CSRS) was utilized to create a 3D circular lamellae construct that mimics the complex AF tissue and which overcomes this translational limitation	Shamsah et al., 2019
Cell therapy	Silk protein-based multilayered, disc-like angle-ply construct		Porcine AF cells and hMSCs	NA	The constructs supported cell proliferation, differentiation, and ECM deposition resulting in AF-like tissue features based on ECM deposition and morphology	Bhunia et al. (2018)
	PTMC and TGF-β3		HASCs	NA	Production of sGAG and collagen was significantly upregulated, but the formed collagen was also oriented and aligned into bundles within the designed pore channels. The differentiated hASCs seeded with fibrin gel were also found to have a comparable sGAG:collagen ratio and gene expression profile as native AF cells	Blanquer et al. (2017)
	PTMC and PU		Human BMSCs	AF rupture repair in a bovine organ culture annulotomy model	It can withstand dynamic loads, restore the height of the intervertebral disc, prevent the protrusion of the nucleus pulposus, and has the ability to differentiate *in situ*	Pirvu et al. (2015)

**TABLE 3:** Summary of various biomaterials for cartilage endplate repair and their functions.

Material type	Material composition	Target cell/gene		*In vivo* experimental model	Repair effect	References
ADSC-TEC			Rat ADSCs	Rat disc degeneration model	Disc height, endplate, and annulus fibrinoid structure were maintained, and age-related biomechanical impairments were reduced	Ishiguro et al. (2019)
NP, CEP, and CPP substrate			Bovine articular chondrocytes and NP cells	NA	A continuous layer of NP tissue was formed and fused with the underlying cartilagenous tissue, which in turn was integrated with the porous CPP to improve the interface between the bone substitute and THE IVD tissue	Hamilton et al. (2006)
Outer AF cells inoculated with angular layer, multilayer PC PU scaffold and articular chondral cells			Bovine outer AF cells and articular chondral cells	NA	The outer AF tissue was integrated with the cartilage and similar to the natural interface. The apparent tensile strength of the *in vitro* interface was also significantly increased	Chong et al. (2020)

## Glossary

AF: Annulus fibrous
ECM: Extracellular matrix
PU: Polyurethane
PC: Polycarbonate
NF-SMS: Nanofiber sponge microspheres
NP: Nuclear pulposus
IPN: Interpenetrating network
sGAGs: Sulfated glycosaminoglycans
PEG: Polyethylene glycol
NP-SCs: Nucleus pulposus-derived stem cells
PFTBA: Perfluorotributylamine
HP: Hyperbranched polymer
AMO: Anti-miR-199a
PLGA: Poly (lactic acid-co-glycolic acid)
NS: Nanospheres
HIF: Hypoxia inducible factor
MSC: Mesenchymal stem cell
MPM: Mixed composite micelle
pDNA: Plasmid DNA
HO-1: Heme oxygenase-1
PCL: Polycaprolactone
DEX: Dextran
PTE: Plastid extract
PECUU: Poly (ether carbonate carbamide) urea
AFSCs: Amniotic fluid-derived stem-cell
DAFM: Decellularized AF matrix
CSRS: Cell sheet rolling system
PLLA: Poly(L-lactic acid) acid
g-DAF-G: Genipin cross-linked acellular AF hydrogel
HDC: High-density collagen gel
DHI: Disk height index
ILM: Interlamellar matrix
PTMC: Poly (trimethylene carbonate)
TGF: Transforming growth factor
BMSCs: Bone marrow mesenchymal stem cells
IGF-1: Insulin-like growth factor-1
PDGF-AB: Platelet-derived growth factor-AB
MDC: Macrophage-derived chemokine
SDF-1: Stromal cell-derived factor-1
TEC: Tissue engineered construct
CPP: Calcium polyphosphate
FGF: Fibroblast growth factor
ASICs: Acid-sensitive ion channels
CTGF: Connective tissue growth factor
